# Mechanobiological modulation: physiological pressure preconditioning boosts the paracrine-driven lymphangiogenic capacity of bone marrow mesenchymal stem cells

**DOI:** 10.3389/fphys.2026.1892485

**Published:** 2026-08-24

**Authors:** Xiaona Zhang, Chang Zhu, Jiang Chen

**Affiliations:** 1 Clinical Research Center for Oral Tissue Deficiency Diseases of Fujian Province & Fujian Key Laboratory of Oral Diseases & Fujian Provincial Engineering Research Center of Oral Biomaterial, School and Hospital of Stomatology, Fujian Medical University, Fuzhou, China; 2 Stomatological Key Laboratory of Fujian College and University & Institute of oral homeostasis & Research Center of Oral Tissue Engineering, Fujian Medical University, School and Hospital of Stomatology, Fujian Medical University, Fuzhou, China; 3 Jincheng People’s Hospital, Jincheng, China; 4 Jincheng Hospital Affiliated to Changzhi Medical College, Jincheng, China

**Keywords:** bone marrow mesenchymal stem cells, lymphangiogenesis, mechanical pressure preconditioning, mechanobiology, paracrine

## Abstract

**Introduction:**

Lymphatic system remodeling and regeneration are essential for tissue repair and homeostasis, and these processes are precisely regulated by both biochemical and biomechanical signals in the tissue microenvironment. The mechanobiological mechanisms governing this process remain largely elusive, particularly in oral and maxillofacial tissues which are constantly exposed to complex mechanical forces. Bone marrow mesenchymal stem cells (BMSCs) hold great promise for regenerative medicine due to their robust paracrine activity, but how mechanical preconditioning modulates their pro-lymphangiogenic paracrine effects has not been systematically investigated.

**Methods:**

Primary rat BMSCs were isolated and characterized, then subjected to mechanical pressure to collect pressure-preconditioned conditioned medium (P-B-CM). Conditioned medium harvested from untreated, routinely cultured BMSCs served as B-CM. Commercially acquired primary lymphatic endothelial cells (LECs) were validated with lymphatic-specific markers, then cultured in B-CM or P-B-CM to assess intergroup functional differences. Proliferation was quantified via CCK-8 and cell cycle assays; migration was examined by wound healing tests and cytoskeleton staining. Transwell permeability assays, VE-cadherin staining, and transmission electron microscopy were used to evaluate barrier integrity. An *in vitro* tube formation assay and ex vivo thoracic duct culture further measured the lymphangiogenic capacity of LECs cultured with different conditioned media.

**Results:**

Compared with the control B-CM group, LECs cultured with P-B-CM exhibited accelerated cell cycle progression and improved cytoskeleton remodeling. Transwell permeability detection, VE-cadherin immunostaining, and TEM observations collectively demonstrated that P-B-CM strengthened intercellular barrier integrity by increasing the length of continuous adherens junctions. Consistent with the *in vitro* cellular findings, ex vivo rat thoracic duct culture further verified that P-B-CM induced more robust lymphatic sprouting, confirming its promotive effects on lymphangiogenesis.

**Conclusion:**

Mechanical pressure preconditioning enhances the pro-lymphangiogenic paracrine activity of BMSCs, optimizes LEC barrier function and lymphatic sprouting, and offers a biomechanical strategy for oromaxillofacial lymphatic regeneration.

## 1 Introduction

The lymphatic system is an indispensable component of the systemic circulatory system, executing pivotal physiological functions including lymph transport, immune modulation, and maintenance of tissue fluid homeostasis. Its structural integrity and functional stability are closely correlated with multiple pathophysiological processes, such as wound repair, inflammation resolution, and tumor metastasis (Flister et al., 2010). Lymphangiogenesis primarily occurs in physiological and pathological remodeling after tissue damage; for instance, this process is reactivated during wound healing and tumor progression (Vaahtomeri et al., 2017; Brunner et al., 2023; Jian et al., 2024; Pollack et al., 2025). Lymphatic dysfunction impedes the recovery of damaged tissues (Sato et al., 2016; Lohr et al., 2026). Lymphatic endothelial cells (LECs) are the core functional cells that construct lymphatic vessels, and their tube-forming capacity serves as a key link in lymphangiogenesis and injury repair. The regulatory mechanisms underlying LEC tube formation have long been a research hotspot in the field of lymphatic biology (Shiiya and Hirashima, 2023; Qin et al., 2024).

Under physiological and pathological conditions, cells reside in a complex mechanical microenvironment. As a crucial mechanical stimulus, pressure is widely involved in tissue injury, inflammatory responses, the tumor microenvironment, organ transplantation and other scenarios (Chien and Tsai, 2023; Melica et al., 2026). Existing studies have demonstrated that pressure stimulation can directly regulate the proliferation, migration and tube-forming abilities of LECs. An abnormal pressure microenvironment triggers disorganized lymphatic structure and functional disorders, thereby exacerbating tissue edema and delaying the progression of injury repair (Planas-Paz and Lammert, 2013; Heron et al., 2026). Therefore, exploring regulatory strategies for LEC tube formation under pressure conditions possesses important clinical significance for optimizing the prognosis of lymphatic-related diseases.

Bone marrow mesenchymal stem cells (BMSCs) are adult stem cells with self-renewal capacity and multidirectional differentiation potential. Owing to their extensive sources, convenient accessibility and low immunogenicity, BMSCs have been widely applied in tissue repair and regeneration research (Li et al., 2025; Wen et al., 2026). Recent studies have revealed that BMSCs secrete a variety of cytokines, growth factors and extracellular matrix components via paracrine effects, which indirectly modulate the biological functions of endothelial cells (including vascular endothelial cells and lymphatic endothelial cells) and promote angiogenesis and lymphangiogenesis (Li et al., 2022; Berry et al., 2025). The facilitatory effect of BMSCs on LEC tube formation under normal physiological conditions has been preliminarily confirmed. Nevertheless, relevant researches are still scarce and no definitive conclusions have been drawn regarding whether BMSCs can maintain their regulatory effects and how they affect the tube-forming function of LECs under a special mechanical microenvironment with pressure intervention.

Based on the above research background, we focused on physiological mechanical pressure, a critical mechanobiological cue in tissue microenvironments, and investigated the effects of conditioned medium derived from normally cultured BMSCs (B-CM) and pressure-preconditioned BMSCs (P-B-CM) on LEC functions. This study aims to clarify the paracrine-mediated regulatory role of BMSCs in lymphangiogenesis under pressure stimulation, fill the current knowledge gap regarding the crosstalk between mechanical microenvironment and stem cell-based lymphatic regeneration, and provide novel theoretical and experimental evidence for the clinical treatment of pressure-related lymphatic injuries.

## 2 Materials and methods

### 2.1 Experimental animals

Sprague-Dawley (SD) rats were purchased from Sibeifu (Beijing) Biotechnology Co., Ltd. Neonatal SD rats aged 3–7 days were used for the isolation of bone marrow mesenchymal stem cells (BMSCs), and 6-week-old male SD rats (body weight: 220 ± 10 g) were used for the isolation of thoracic ducts. All animal experimental procedures were approved by the Animal Ethics Committee of Fujian Medical University (approval number: IACUC FJMU 2025-0012).

### 2.2 Isolation, flow cytometric identification, and osteogenic/adipogenic differentiation capacity assay of BMSCs

Neonatal SD rats were deeply anesthetized by inhalation of 3–5% isoflurane in oxygen, followed by rapid decapitation for euthanasia. Bilateral femurs and tibias were aseptically dissected, and the bone marrow cavity was flushed with BMSC complete medium (BLDM-03011, OriCell, Guangzhou, China) supplemented with 1% penicillin-streptomycin solution (P1400, Solarbio, Beijing, China). The cell pellet was collected by centrifugation, then inoculated and cultured in a 5% CO_2_ incubator at 37 °C. The culture medium was refreshed 3 days after inoculation. Subculture was performed when the confluency of adherent cells reached over 90%, and cells at passage 3–5 were used for all subsequent experiments.

Passage 3 BMSCs were harvested by digestion with 5% trypsin (T1350, Solarbio, Beijing, China), resuspended in PBS buffer, and adjusted to a concentration of 1 × 10^6^ cells/mL. Then 100 μL of the cell suspension was incubated with FITC-conjugated anti-rat CD45 antibody (E-AB-F1227C, Elabscience, Wuhan, China), APC-conjugated anti-mouse/rat CD29 antibody (E-AB-F1309E, Elabscience, Wuhan, China), PerCP-Cyanine5.5-conjugated anti-rat CD90 antibody (E-AB-F1226J, Elabscience, Wuhan, China), PE-conjugated anti-rat CD11b/c antibody (201807, BioLegend, San Diego, USA), or PBS in the dark for 30 min, with gentle shaking during incubation to ensure sufficient binding. The experimental group was treated with the above four specific antibodies. After incubation, cells were collected by centrifugation at 1000 r/min for 5 min at 4 °C, washed twice with PBS, and finally resuspended in 500 μL PBS. Flow cytometry analysis was performed on a FACSMelody flow cytometer (BD, USA).

For osteogenic differentiation, BMSCs were cultured in osteogenic induction medium (RAXMX-90021, OriCell, Guangzhou, China) for 21 days, with medium changed every 3 days, and stained with Alizarin Red S (ALIR-10001, OriCell, Guangzhou, China). For adipogenic differentiation, BMSCs were cultured in adipogenic induction medium (Cyagen Biosciences) for 21 days, and stained with Oil Red O (OILR-10001, OriCell, Guangzhou, China).

### 2.3 Identification of LECs (immunofluorescence, western blot)

Human lymphatic endothelial cells (LECs, primary) were purchased from Otwo Biotech (Cat# HTX2215, Shenzhen, China) and cultured in endothelial cell medium (Sciencell, Cat# 1001, USA). LECs at passages 3–6 were used for all experiments.

For immunofluorescence staining, LECs were seeded on coverslip and cultured until reaching 80% confluence. Cells were fixed with 4% paraformaldehyde (P1110, Solarbio, Beijing, China) for 10 min, permeabilized with 0.3% Triton X-100 (T8200, Solarbio, Beijing, China) in PBS for 10 min, and then blocked with 10% normal goat serum (SL038, Solarbio, Beijing, China) at room temperature for 1 h. After removing the blocking solution (without washing), the cells were incubated with primary antibodies overnight at 4°C: rabbit anti-LYVE-1 (1:100, 28321-1-AP, Proteintech, Wuhan, China) and rabbit anti-Podoplanin (1:100, 11629-1-AP, Proteintech, Wuhan, China). Subsequently, the cells were incubated with the corresponding secondary antibody (1:100, RGAR004, Proteintech, Wuhan, China) for 45 min at room temperature. After counterstaining with DAPI for 5 min and washing with PBS, the coverslip was mounted with anti-fade mounting medium (S21110, Solarbio, Beijing, China) onto glass slides, imaged using a laser scanning confocal microscope (LEICA/SP8, Germany).

Total protein was extracted from LECs using RIPA lysis buffer (Beyotime, Cat# P0013J, China) containing 1 mM PMSF (Solarbio, Cat# P0100, China). Protein concentration was measured with a BCA protein assay kit (BL2440A, Labgic, Anhui, China). Following gel electrophoresis, the proteins were transferred to a PVDF membrane and blocked with 5% skimmed milk powder for 1 hour. Diluted primary antibody solutions were then added: Anti-GAPDH (81640-5-PR, Proteintech, Wuhan, China), Anti-LYVE-1 (1:1000, 28321-1-AP, Proteintech, Wuhan, China) and Anti-Podoplanin (1:1000, 11629-1-AP, Proteintech, Wuhan, China). The membrane was incubated overnight at 4 °C. After the primary antibody incubation, the membrane was thoroughly washed with TBST. A diluted secondary antibody solution (Anti-Rabbit IgG (H+L): RGAR001, Proteintech, Wuhan, China) was then added and incubated at room temperature for 1 hour. Following this incubation, the membrane was again thoroughly washed with TBST. Finally, the ECL luminescence working solution (PK10003, Proteintech, Wuhan, China) was prepared, and the protein bands were developed using a gel imaging system (Chemidoc, Bio-Rad, USA).

### 2.4 Preparation of BMSC-derived conditioned media and screening of optimal mechanical pressure parameters

**Figure 1:**
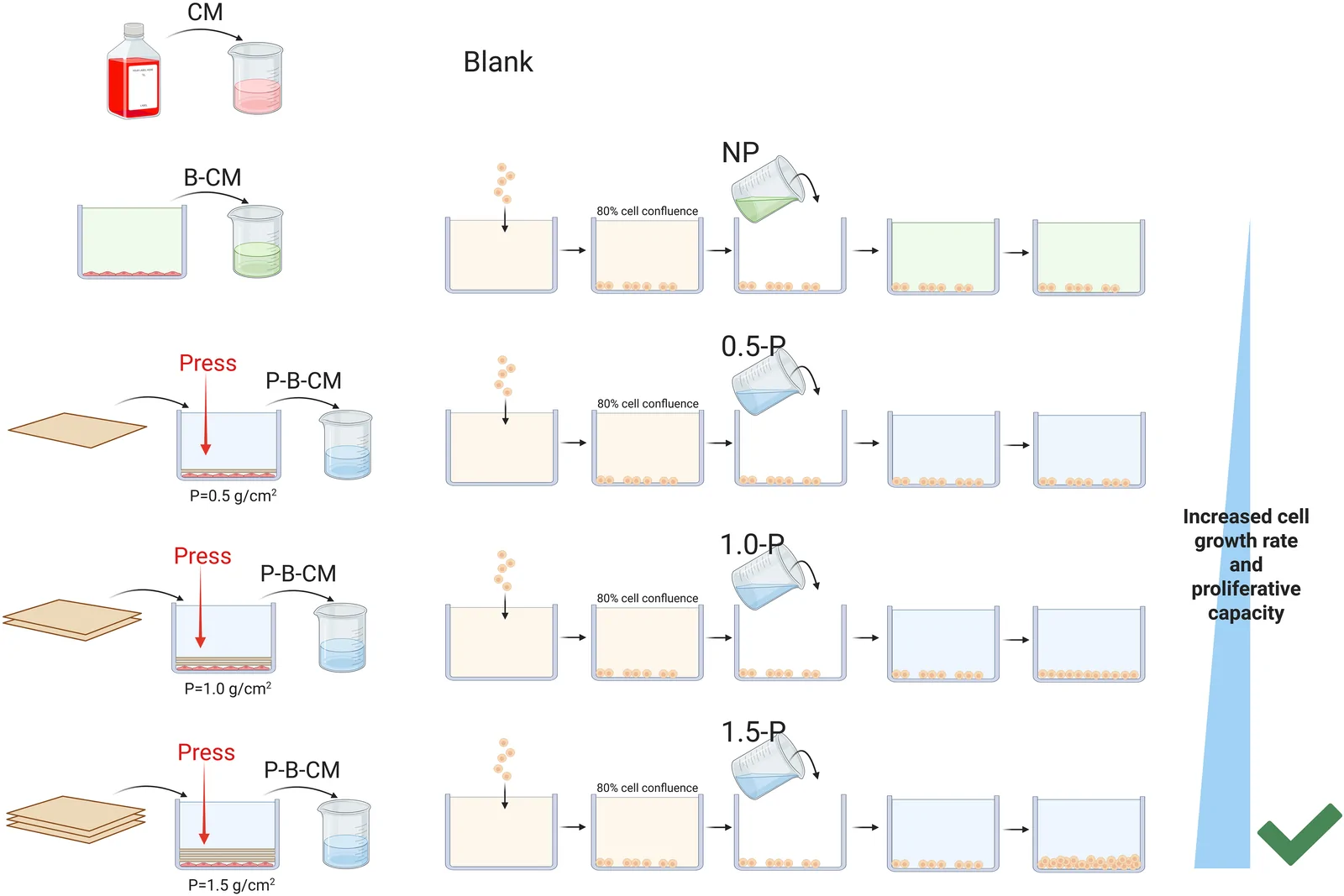
Schematic diagram of extraction of conditioned media from each group and determination of optimal pressure.

To guarantee consistent culture conditions across all groups, identical BMSC seeding density, culture dish area, medium volume and incubation duration were strictly maintained throughout the experiment. When BMSCs reached approximately 80% confluence, the original culture medium was discarded, and the cells were rinsed with PBS, then replaced with endothelial cell medium (#1001, Sciencell, USA) supplemented with 0% fetal bovine serum (FBS). According to the methods described in previous literature (Albogha and Takahashi, 2019; Jin et al., 2020; Brockhaus et al., 2021; Moga et al., 2024; Zheng et al., 2024), gradient mechanical pressure was applied using glass slides of different thicknesses: BMSCs were subjected to 0.5 g/cm² (0.0490kPa), 1 g/cm²(0.0981kPa), and 1.5 g/cm²(0.1471kPa) of pressure for 12 h, respectively. The collected conditioned medium samples were centrifuged at 2500 rpm for 20 min at 4 °C to remove cellular debris. All collected conditioned media were normalized according to the count of viable BMSCs in each culture plate to eliminate differences in total secreted products among groups. The clarified supernatants were subpackaged and preserved at −80 °C for subsequent experiments. For subsequent LEC culture experiments, we set five groups with unified abbreviations: Blank group (LECs cultured with pure endothelial cell medium); NP group (LECs cultured with CM collected from non-pressure BMSCs, defined as B-CM); 0.5-P group (LECs cultured with CM from BMSCs treated with 0.5 g/cm² pressure); 1.0-P group (LECs cultured with CM from BMSCs treated with 1 g/cm² pressure); 1.5-P group (LECs cultured with CM from BMSCs treated with 1.5 g/cm² pressure). LECs from the group with the optimal proliferation effect were selected for cell viability assay and live/dead cell staining. The experimental procedure is shown in **Figure 1**.

### 2.5 CCK8, live-dead assay and reactive oxygen species detection

Each group of conditioned media was co-cultured with LECs, with five replicate wells established for each group. The culture medium was discarded at 12, 24, 48, and 72 hours. Subsequently, the wells were incubated with basal medium containing 10% Cell Counting Kit-8 (BS350B, Labgic, Beijing, China) for 1 hour, after which the optical density (OD) of each well was measured using a microplate reader (Infinite plex, TECAN, China) at A450. Statistical analyses were conducted among the groups.

When BMSCs were subjected to a pressure of 1.5 g/cm² for 0, 6, 12, and 24 hours, Annexin V-mCherry and SYTOX Green (C1070S, Beyotime, China) staining was performed, followed by observation and photography under an inverted fluorescence microscope (LEICA/BMi8).

Intracellular ROS levels in BMSCs under different compressive pressure (0, 0.5, 1.0, 1.5, 2.0, 2.5 g/cm²) were detected using the DCFH-DA fluorescent probe kit (S0033S, Beyotime, China) following the manufacturer’s instructions.

### 2.6 Cell cycle analysis and cell migration assay

Three culture medium systems were prepared in this study, and the same three-group system was uniformly applied to all subsequent cellular functional experiments. The groups were defined as follows:

- Blank group: standard endothelial medium processed through identical incubation, centrifugation, and filtration procedures as conditioned medium, but without BMSC incubation.
- NP group: conditioned medium collected from unstimulated BMSCs, abbreviated as B-CM.
- P group: conditioned medium derived from BMSCs treated with 1.5 g/cm² compressive force, abbreviated as P-B-CM.

LECs were seeded into 6-well culture plates and cultured until the cell confluence reached approximately 80%. The culture medium was then replaced with the three types of medium described above (Blank, NP, P) for corresponding groups, and cells were incubated for another 24 h. Subsequently, LECs were digested and harvested, followed by two washes with pre-cooled PBS. Cell pellets were fixed in pre-cooled 70% ethanol at 4 °C overnight. On the next day, fixed cells were rinsed with PBS again, resuspended in propidium iodide (PI) staining solution (C1052, Beyotime, China) supplemented with RNase A, and incubated in the dark at room temperature for 30 min. Flow cytometry was utilized to detect intracellular DNA content, and the proportion of cells distributed in G1, S and G2 phases was quantified by the Flowjo software.

The scratch wound healing assay was applied to evaluate LEC migratory capacity. Vertical reference lines were drawn on the bottom of 12-well plates, followed by seeding 2×10^5^ LECs per well. After overnight incubation until cells reached 90%–95% confluence, a sterile 200 μL pipette tip was used to create linear scratches perpendicular to the reference lines in each well. Floating detached cells were washed off with PBS three times. The medium was replaced with the three types of conditioned medium described above for corresponding groups, and cells were cultured continuously for 24 h. Cells were incubated at 37 °C under 5% CO_2_. Bright-field images of pre-marked fixed fields were captured at 12 h and 24 h post-scratching under an inverted microscope (AE2000, Motic, China) at 10× magnification. Fiji ImageJ-win64 software (National Institutes of Health, USA) was used to quantify scratch coverage area variations for the statistical assessment of cell migration.

### 2.7 Effect of P-B-CM on LEC adhesion function

96-well plates were coated with 1% gelatin (C0316, Beyotime, China) and incubated overnight at 4°C. After washing 3 times with PBS, the plates were blocked with 1% BSA (A8010, Solarbio, China) at 37°C for 1 h, followed by another 3 washes with PBS. LECs cultured in corresponding media for 1 d were seeded into the pre-coated 96-well plates at a density of 5×10^3 cells/well. After incubation at 37°C for 1 h, non-adherent cells were removed by gentle washing 3 times with PBS. Cells were fixed with 4% paraformaldehyde for 15 min, washed 3 times with PBS, stained with 0.1% crystal violet (C8470, Solarbio, China) for 5 min, and washed 3 times with PBS. Images were captured under a microscope (AE2000, Motic, China).

### 2.8 Transwell permeability assay

The upper chamber of the Transwell (3470, Corning, USA) insert was pre-wet with 100 μL serum-free medium for 10 min, then 100 μL LECs at a density of 2×104 cells/mL were seeded. The lower chamber was filled with 600 μL complete medium. The next day, the medium was replaced with corresponding conditioned medium for each group. After culturing for 3–5 days to form a confluent monolayer, the upper chamber medium was replaced with serum-free medium containing 0.1 mg/mL dextran (ST2930, Beyotime, China), and the lower chamber was filled with 600 μL conditioned medium. Fluorescence changes in the lower chamber at 500 nm were detected at different time points (0.5 h, 1 h) using a microplate reader (LEICA/BMi8, Germany).

### 2.9 Immunofluorescence staining for cytoskeleton and cell junctions

Phalloidin staining was performed: LECs were seeded on 24-well plates with coverslips. When cell density reached 80%, the medium was replaced with conditioned medium and cultured for 1 d. Then, 4% paraformaldehyde (P1110, Solarbio, Beijing, China) was added to each well for fixation for 15 min, followed by 3 washes with PBS. After permeabilization with 0.1% Triton X-100 (T8200, Solarbio, Beijing, China) for 10 min, the cells were washed 3 times with PBS. Actin-Tracker Green-488 (C2232, Beyotime, China) was added and incubated at room temperature for 15 min. The cells were washed 3 times with PBS (3 min each). In the dark, 100 μL DAPI (2 μg/mL) was added to each well and incubated at room temperature for 5 min. After 3 washes with PBS (5 min each), the coverslips were mounted with anti-fade mounting medium (S21110, Solarbio, Beijing, China) onto glass slides, imaged using a laser scanning confocal microscope (LEICA/SP8, Germany).

After fixing the cells following the above steps, add Anti-VE-Cadherin (27956-1-AP, Proteintech, Wuhan, China) and incubate overnight at 4°C. The subsequent procedures are the same as those described in Section 2.3.

### 2.10 Transmission electron microscopy observation

Third-passage LECs were seeded in 6 cm culture dishes. When the cell density reached approximately 80%, the medium was replaced with conditioned medium. After 1 day of culture, most of the culture medium was aspirated, leaving about 1 mL in the dish. Cells were harvested by scraping with a cell scraper at a 45° angle in a single direction, and the cell suspension was transferred to a 1.5 mL centrifuge tube. Cells were pelleted by centrifugation at 2000 rpm for 8 min. The supernatant was aspirated, and room-temperature 2.5% glutaraldehyde fixative (P1126, Beyotime, China) was slowly added along the tube wall to maintain the pellet. The pellet was fixed at room temperature for 30 min, then stored at 4°C for 24 h. After dehydration and embedding, ultrathin sections (EMUC7, LEICA, Germany, 60–70 nm) were prepared, stained with uranyl acetate (1261209, SPI, USA) and lead citrate (02601, SPI, USA), and observed under a transmission electron microscope (JEM-1400, JEOL, Japan) at 80 kV. Under TEM, continuous intercellular adherens junctions between LECs were manually traced and measured via ImageJ software. We calculated intact zipper-shaped adherens junction segments.

### 2.11 Tube formation assay

Pipettes and tips were pre-chilled, and all operations were performed on ice. 20 μL of Matrigel (A1413202, Gibco Geltrex, USA) was added to the center of each well in a 24-well plate and spread evenly from the inside out with a pipette tip, avoiding bubble formation and contact with the sealing film on the well walls. The plate was incubated at 4°C overnight to allow the Matrigel to spread naturally. The next day, the plate was transferred to a 37°C incubator to solidify the Matrigel for 1 hour. In a biosafety cabinet, digested cells were seeded onto the Matrigel at a density of 1×10^5^ cells per well with corresponding media for the Blank group, NPgroup, and P group, then incubated in the culture chamber. Images were captured under a microscope at 4× magnification at 2 h, 6 h, and 10 h of culture. The total length of vascularization, number of tubes, and number of branch points were quantified using Fiji ImageJ-win64 software.

### 2.12 Quantification and analysis of thoracic duct ring sprouting

Six-week-old SD rats were deeply anesthetized by intraperitoneal injection of 2% sodium pentobarbital solution at a dose of 50 mg/kg body weight. After achieving deep anesthesia (confirmed by the absence of withdrawal reflexes), the rats were euthanized via cervical dislocation. Rats were disinfected with 75% ethanol, and underwent thoracotomy to locate and isolate the thoracic duct (on the surface of the spinal column, posterior to the esophagus, slightly to the right of the midpoint between the azygos vein and thoracic aorta). The isolated thoracic duct was placed in pre-cooled medium (#1001, Sciencell, USA) containing double antibiotics and cut into 1 mm tissue fragments for subsequent use. All operations were performed on ice. 150 μL of pre-cooled Matrigel was added to each well of a 24-well plate, and the thoracic duct tissue fragments were embedded in it. After being placed in the incubator for 20 min to allow solidification, 300 μL of complete lymphatic endothelial cell medium (#1001, Sciencell, USA) was added, and the culture was maintained for 2–3 days. Once LECs migrated outward, the medium was replaced with supernatants from the Blank, NP and P group. Images of the same field of view were captured at 0 h, 24 h, and 48 h, and the sprouting area growth rate and sprouting density increase rate were analyzed using Fiji ImageJ-win64 software.

### 2.13 Enzyme-linked immunosorbent assay detection of VEGF-C and VEGF-D

Conditioned medium (B-CM and P-B-CM) was collected following the procedures described in Section 2.4. The secretion levels of two core lymphangiogenic master regulators (VEGF-C and VEGF-D) were quantified using commercial ELISA kits (AYP-R0575; SYP-R0262, UpingBio, China) following the manufacturer’s protocols. Briefly, normalized Conditioned medium samples were loaded into pre-coated microplates, and absorbance values were read via a microplate spectrophotometer at the specified wavelength. Protein concentrations of VEGF-C and VEGF-D were calculated based on the standard curve generated in parallel.

### 2.14 Statistical analysis

Data were analyzed using GraphPad Prism 10.0.2 software (GraphPad, La Jolla, CA, USA). All individual experiments were repeated at least N = 3 times per experiment and each experiment was replicated at least three times. For comparisons among multiple groups, one-way analysis of variance (ANOVA) was performed, followed by Dunnett’s *post-hoc* test. For comparisons between two groups, unpaired Student’s t-test was used. A p-value < 0.05 was considered statistically significant.

## 3 Results

### 3.1 Successful isolation and identification of BMSCs and verification of LECs

**Figure 2:**
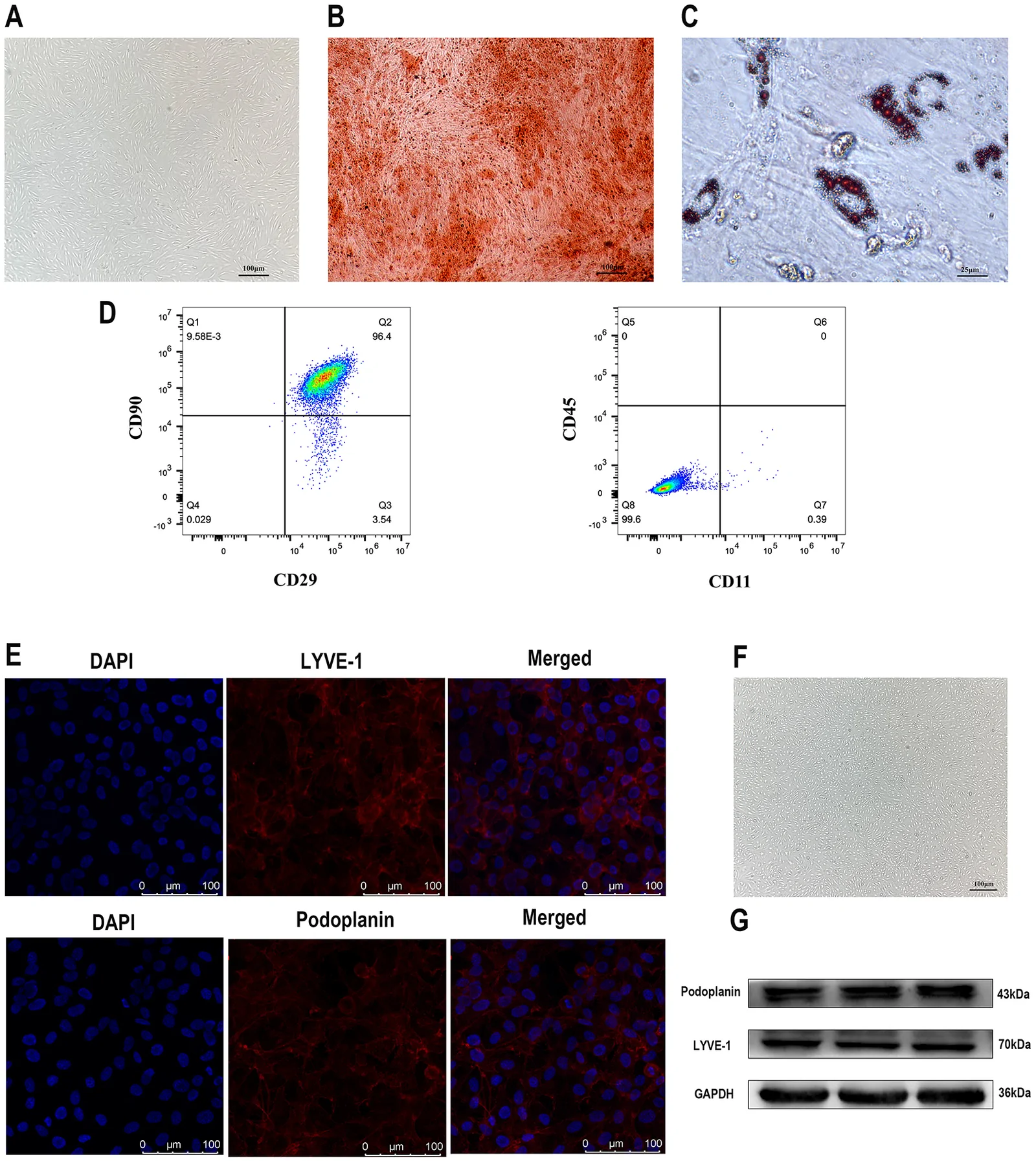
Identification of BMSC and LEC. **(A)** Microscopic morphology of BMSCs; **(B)** BMSCs osteogenic differentiation with Alizarin Red staining; **(C)** BMSCs adipogenic differentiation with Oil Red O staining; **(D)** BMSCs flow cytometry; **(E)** LYVE-1 and Podoplanin immunofluorescence staining (Scale bar: 100μm); **(F)** LECs microscopic morphology; **(G)** Western blotting of LYVE-1 and Podoplanin.

Cells isolated from the bone marrow cavity of neonatal SD rats exhibited a spindle-shaped, adherent growth pattern, forming a typical whirlpool-like arrangement at confluence (**Figure 2A**), consistent with the morphological characteristics of BMSCs. Osteogenic and adipogenic differentiation induction and staining confirmed the multipotent differentiation potential of the cells (**Figures 2B, C**). Flow cytometry results showed that the double-positive rate of BMSC surface markers CD29 and CD90 was 96.4%, and the double-negative rate of hematopoietic markers CD45 and CD11b was 99.6% (Amado et al., 2005) (**Figure 2D**), which conformed to the immunophenotypic characteristics of BMSCs, indicating that high-purity BMSCs suitable for subsequent experiments were successfully isolated.

Lymphatic vessel endothelial hyaluronan receptor 1 (LYVE-1) (Banerji et al., 1999) and podoplanin (also known as D2-40) (Breiteneder-Geleff et al., 1999) are specific markers of LECs. Immunofluorescence staining showed that both markers were uniformly expressed in LECs (**Figure 2E**). Combined with morphological observation (**Figure 2F**) and Western blot verification (**Figure 2G**), it was confirmed that the purchased LECs were of qualified purity and could be used for subsequent experiments.

### 3.2 BMSC secretions under pressure promote LEC proliferation

**Figure 3:**
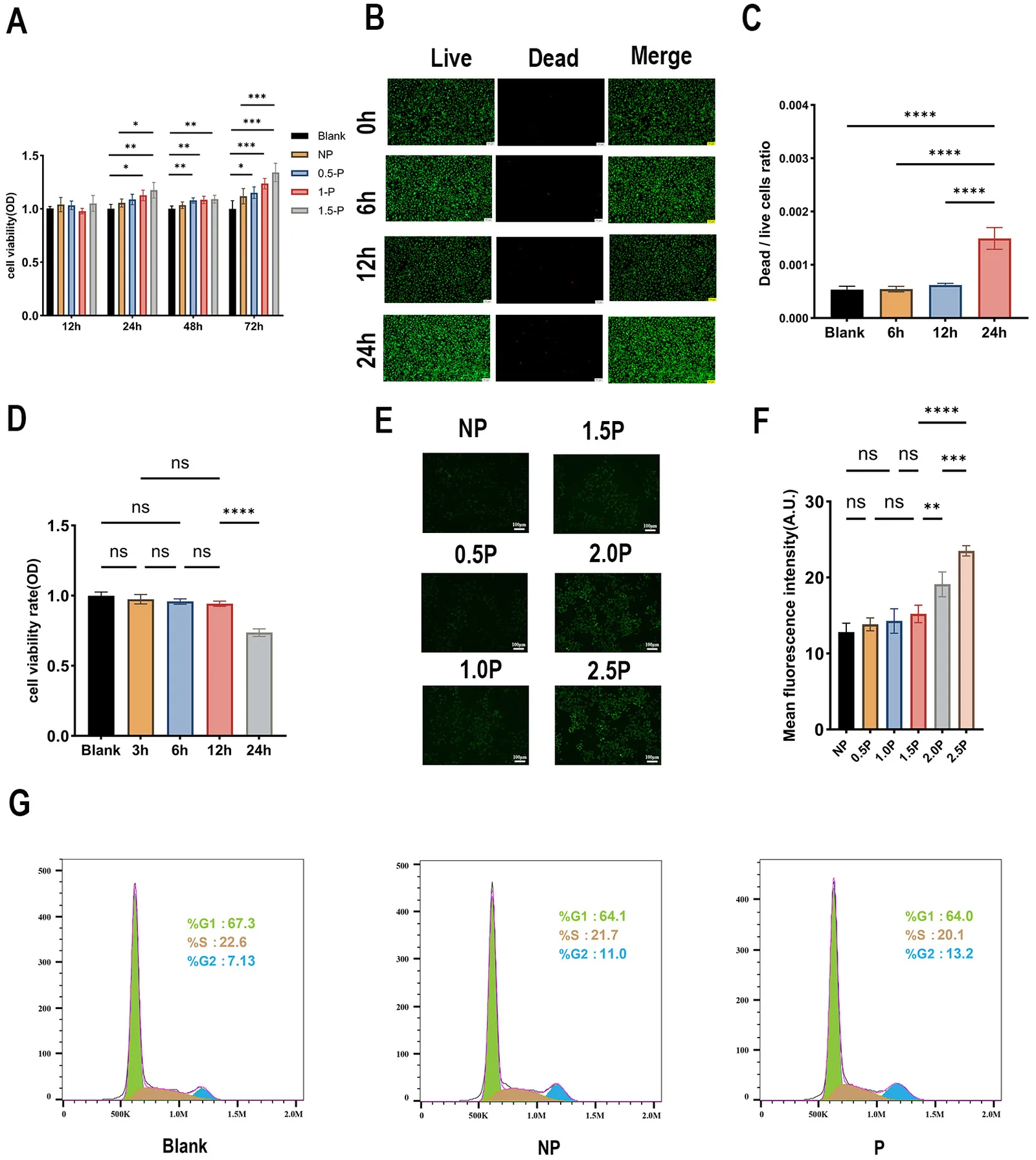
BMSC secretions under pressure promote LEC proliferation. **(A)** CCK8 determination of LECs in each group of conditioned media; **(B)** BMSCs were stained(red: dead cells; green: live cells) and quantitatively analyzed **(C)** for viability at different times under a pressure of 1.5 g/cm^2^ (Scale bar: 100μm); **(D)** BMSCs are measured with CCK8 at different times under a pressure of 1.5 g/cm^2^; **(E)** ROS immunofluorescence staining images and quantitative analysis **(F)** of intracellular reactive oxygen species levels in compressed BMSCs; **(G)** Cell cycle determination of LECs in each group (n = 5, data are expressed as mean ± SEM. *P ≤ 0.05, **P ≤ 0.01, ***P ≤ 0.001, ****P ≤ 0.0001). The subpanels follow the experimental sequence of gradient compressive medium screening, BMSC biosafety verification and subsequent LEC proliferation validation.

CCK-8 assay was performed to evaluate the effect of BMSC secretions collected under different pressure conditions (0.5,1, 1.5 g/cm²) on LEC viability. The results demonstrated that pressure-treated BMSC secretions significantly enhanced LEC proliferation, with 1.5 g/cm² exerting the strongest promotional effect (**Figure 3A**). Live/dead cell staining and CCK-8 assay confirmed that 1.5 g/cm² pressure treatment for 12 hours had no significant cytotoxicity to BMSCs (**Figures 3B–D**). DCFH-DA reactive oxygen species (ROS) staining verified obvious ROS accumulation and cell damage only appeared at the super-physiological 2.0 g/cm² and 2.5 g/cm² groups (**Figures 3E, F**). Considering its prominent pro-proliferative activity and non-cytotoxic property within the physiological range, 1.5 g/cm²/12 h was selected as the representative compressive condition for subsequent experiments. Cell cycle analysis showed that the P group had the highest proportion of S and G_2_ phase cells (**Figure 3G**), indicating activated cell proliferation. The CV (6.93%) and RMSD (1.23) values verified the reliability of the data.

### 3.3 P-B-CM promotes LEC migration and cytoskeletal remodeling without affecting cell adhesion

**Figure 4:**
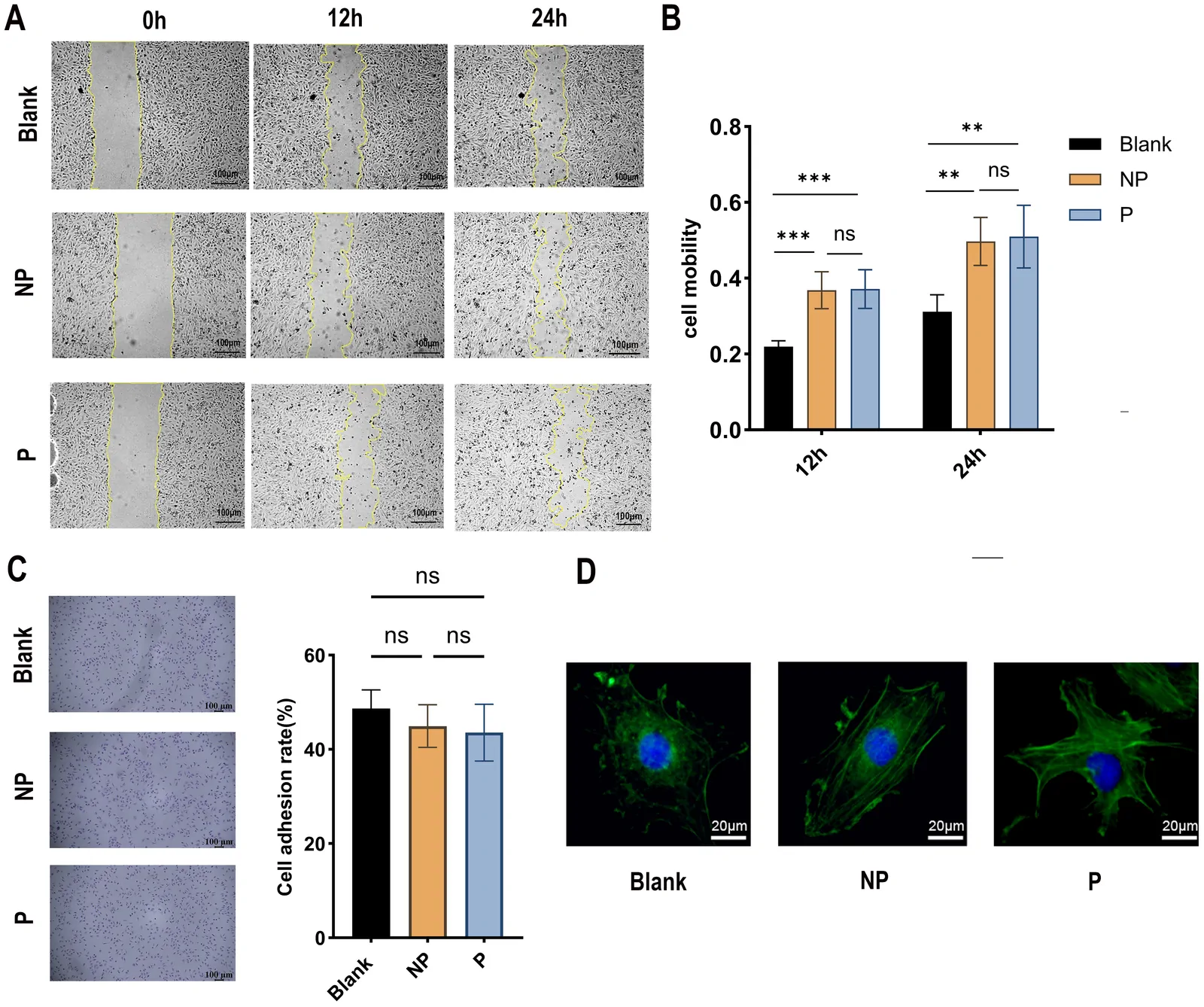
P-B-CM promotes LEC migration and cytoskeletal remodeling without affecting cell adhesion. **(A)** Scratch images at different time points for each group (Scale bar: 100μm); **(B)** Analysis of scratch closure rate; **(C)** Stained images of LECs adhered cells under various conditions and Analysis of LECs adhesion rate under different conditions; **(D)** Cytoskeleton staining of LECs in each group (n = 3, data are expressed as mean ± SEM. *P ≤ 0.05, **P ≤ 0.01, ***P ≤ 0.001).

The scratch wound migration assay revealed that both P-B-CM (P group) and untreated B-CM (NP group) markedly accelerated LEC migratory capacity relative to the Blank group at 12 h and 24 h (**Figures 4A, B**). Minimal migratory differences were observed between the P and NP groups, whereas the Blank group exhibited distinctly slower wound closure compared with the two conditioned medium-treated groups. The cell adhesion assay revealed no significant difference in adhesion rate among the three groups (**Figure 4C**). Rhodamine-phalloidin staining demonstrated that P-B-CM induced prominent F-actin expression, elongated cell morphology, and abundant filopodia in LECs, indicating cytoskeletal remodeling (**Figure 4D**).

### 3.4 P-B-CM enhances barrier integrity in LECs

**Figure 5:**
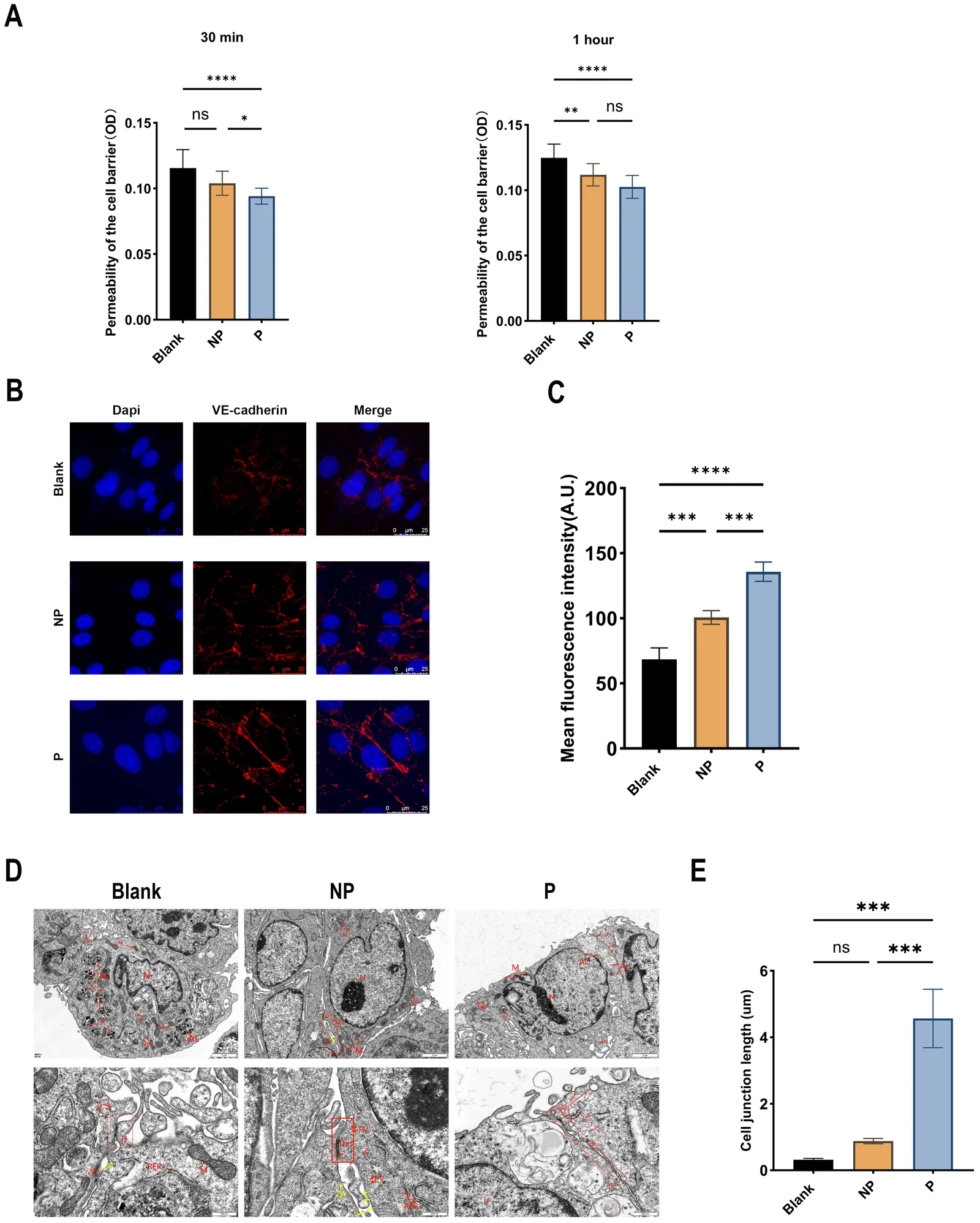
P-B-CM enhances barrier integrity in LECs. **(A)** Determination of permeability of LECs in each group; **(B)** Representative images of VE-cadherin immunofluorescence staining for each group of LEC and quantitative analysis **(C)** of VE-cadherin fluorescence intensity; **(D)** Representative images of LECs in each group under transmission electron microscopy; **(E)** Quantitative analysis of lymphatic adherens junctions in each group. nuclei (N), mitochondria (M), endoplasmic reticulum (ER), Golgi apparatus (GA), autophagic lysomes (AL), tight junctions (TJ), intermediate junctions (ZA), microfilaments (MF) and endocytic vesicles (PV) (n=3, data are expressed as mean ± SEM, *P ≤ 0.05, **P ≤ 0.01; ****P ≤ 0.001).

The endothelial permeability assay showed that P-B-CM significantly reduced LEC permeability in a time-dependent manner, with the P group exhibiting the lowest permeability at 30 min and 1 h (**Figure 5A**). Immunofluorescence staining revealed that VE-cadherin expression was significantly upregulated in the P group compared with the NP and Blank groups (**Figures 5B, C**). Transmission electron microscopy demonstrated that P-B-CM treatment improved the structural integrity of intercellular junctions, with the P group showing continuous junctions, abundant organelles, and concentrated pinocytotic vesicles (**Figure 5D**). Quantitative analysis confirmed that the lymphatic adherens junction length was significantly higher in the P group than in the NP and Blank groups (**Figure 5E**).

### 3.5 P-B-CM enhances lymphatic tube formation

**Figure 6:**
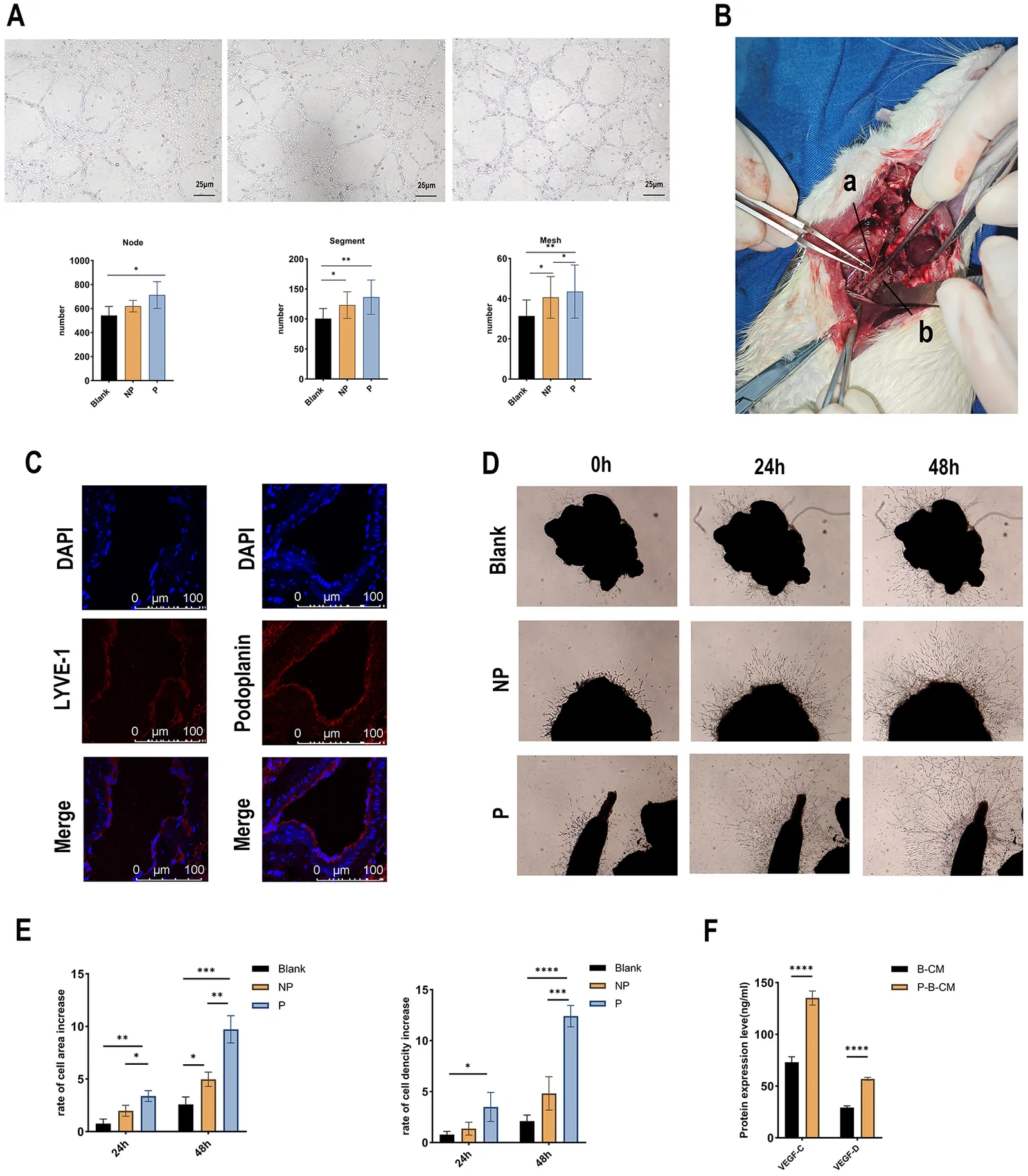
P-B-CM Enhances Lymphatic Tube Formation. **(A)** Under the microscope images of LECs in each group and Quantitative analysis of node, junction, and mesh of tubes treated with different groups; **(B)** Extract images of the tissue separation process of the thoracic duct (a: thoracic duct; b: azygos vein); **(C)** LYVE-1 and Podoplanin immunofluorescence staining; **(D)** Generation images of isolated thoracic duct lymphatic rings in each group at different time points; **(E)** Analysis of lymphatic sprouting increased rate and sprouting density increased rate; **(F)** ELISA quantification of secreted VEGF-C and VEGF-D levels in collected conditioned medium (n=3, data are expressed as mean ± SEM.* P ≤ 0.05; ** P ≤ 0.01; ***P ≤ 0.001; **** P ≤ 0.0001).

Compared with the Blank group, supplementation with B-CM promoted LEC tube formation (**Figure 6A**), and this effect was further enhanced by P-B-CM. Analysis of tube formation parameters showed that the P group exhibited an increasing trend in node number and segment length compared with the NP group, with the highest number of meshes, followed by the NP group and the Blank group.

To further validate the results, an organoid culture model was used: thoracic duct tissues were isolated from SD rats (**Figure 6B**), verified by lymphatic-specific markers (**Figure 6C**), and then cultured in the corresponding media of the Blank, NP, and P groups to evaluate lymphangiogenesis at different time points (**Figure 6D**). Evaluation of tube density and sprout growth demonstrated that lymphangiogenesis in the P group was significantly stronger than that in the NP group at 24 h and 48 h, with the NP group outperforming the Blank group (**Figure 6E**).

Consistent with the lymphatic tube formation phenotypes above, ELISA quantification further revealed that the secretion of two core lymphangiogenic regulators VEGF-C and VEGF-D was markedly upregulated in P-B-CM relative to untreated B-CM (**Figure 6F**). This evidence indicated that mechanical compressive stimulation elevated the paracrine release of lymphangiogenic cytokines from BMSCs, which serves as the key upstream mediator facilitating the enhanced lymphangiogenic capacity of LECs.

## 4 Discussion

In bone trauma regions such as fracture sites and implant surgical areas, appropriate mechanical pressure facilitates bone tissue regeneration (Salhotra et al., 2020), accompanied by vascular remodeling and neogenesis (Gan et al., 2025; Long et al., 2025; Wu X. et al., 2025). In this study, we treated LECs and thoracic duct rings with P-B-CM, and demonstrated that P-B-CM exerts a positive regulatory effect on the biological functions of LECs, including proliferation, migration, adhesion, permeability, morphological alteration, and tube-like structure formation.

Referring to published *in vitro* compressive models simulating mechanical microenvironment of oral, maxillofacial and peri-implant tissues, three compressive gradients of 0.5 g/cm² (0.049 kPa), 1.0 g/cm² (0.098 kPa) and 1.5 g/cm² (0.147 kPa) were established for screening in the present study. These loading magnitudes are widely adopted within oral biomechanics literature to mimic physiological compressive stimuli around implant-bone interfaces (Albogha and Takahashi, 2019; Jin et al., 2020; Brockhaus et al., 2021; Moga et al., 2024; Zheng et al., 2024). A series of cellular assays, including CCK-8 proliferation detection, Live/Dead cell staining and ROS fluorescence staining, jointly confirmed that 1.5 g/cm² compressive treatment for 12 h was suitable for our experimental system. Under this loading parameter, the conditioned medium derived from compressed BMSCs produced the strongest promotive effect on LEC proliferation, while no obvious cytotoxicity or hypoxic damage was observed in BMSCs, with cell viability maintained above 90%. This result is consistent with previous published literature, which reported that the cell survival rate was lower than 80% when the pressure exceeded 2 g/cm², while it remained above 80% at a pressure of ≤1 g/cm² (Shen et al., 2023). In our study, the cell survival rate of the 1.5 g/cm² pressure group was higher than 90%, which was superior to the results in the aforementioned report. This discrepancy may be attributed to differences in the duration of pressurization and cell culture system.

Lymphangiogenesis relies on sequential processes including the proliferation, migration, and morphological remodeling of endothelial cells. Elevated proliferative and migratory capacities, along with morphological alterations of LECs, emerge as early as the initial phase of lymphangiogenesis. Our results demonstrated that incubation with P-B-CM significantly elevated the proliferative and migratory abilities of LECs, accompanied by an increased percentage of cells in the S and G2 phases of the cell cycle. No significant difference was observed in the cell adhesion assay, while cytoskeleton staining revealed that LECs in the P group exhibited a more elongated morphology, abundant pseudopodia, and irregular cell margins, indicating that the cells were in a functionally active state. This finding is consistent with previous research conclusions on the regulation of vascular endothelial cells by mechanical pressure (Farjood et al., 2020; Xie et al., 2020; Shen et al., 2023; Li et al., 2024; Wu Y. et al., 2025).

VE-cadherin is an endothelial cell-specific adhesion molecule closely associated with lymphatic vessel maturation and stabilization. Initial lymphatic capillaries feature discontinuous button-like junctions to facilitate interstitial fluid uptake, during the maturation phase of lymphatic vessels, upregulated VE-cadherin facilitates the formation of continuous intercellular adherens junctions, which are essential to maintain lymphatic endothelial barrier integrity and limit excessive paracellular leakage (Baluk et al., 2007; Hägerling et al., 2018; Nakashima and Hong, 2022). Our immunofluorescence results showed that intercellular VE-cadherin expression was increased in LECs of the P group, Transmission electron microscopy (TEM) results provided ultrastructural evidence supporting this maturation phenotype: LECs in the P group had abundant organelles and elongated adherens junctions, which reflected vigorous cellular metabolism and intact endothelial barrier structure. The permeability assay further supplied quantitative functional verification: the permeability of LEC monolayers in the P group was decreased, consistent with the upregulated VE-cadherin and elongated adherens junctions shown in immunofluorescence and TEM results. This regulatory pattern is similar to that of the vascular endothelial barrier (Lampugnani et al., 2018), suggesting that lymphatic barrier function exerts a positive effect on the development and maturation of lymphatic vessels.

To better mimic the *in vivo* physiological microenvironment, we further validated our findings using three-dimensional (3D) *in vitro* models. First, in the Matrigel-based tube formation assay, compared with the NP group, LECs in the P group exhibited a significant increase in the number of nodes, meshes, and segments of tube-like structures at both 6 h and 10 h, which is consistent with previous reports that mechanical pressure promotes tube-like structure formation in vascular endothelial cells (Jie et al., 2025). This observation reflects the effect of compressive stress on the early sprouting and proliferation stage of lymphangiogenesis. Second, the ex vivo isolated thoracic duct ring culture model is a well-established bridging model between *in vitro* cellular experiments and *in vivo* animal studies (Baker et al., 2011; Moleiro et al., 2017; Moog et al., 2020; Wang et al., 2020). Our results showed that P-B-CM significantly increased the lymphatic sprouting rate and sprout proliferation density of thoracic duct rings. Notably, such pro-lymphangiogenic effects act synergistically with the maturation-promoting phenotype described above: mechanical stimulation not only triggers initial lymphatic endothelial sprouting and tube formation, but also upregulates VE-cadherin to remodel cell junctions and stabilize newly formed lymphatic vessels. The above findings dually confirmed at both the cellular and tissue levels that mechanical pressure effectively promotes LEC-mediated lymphangiogenesis while facilitating subsequent lymphatic vessel maturation.

VEGF-C and VEGF-D are well-established master paracrine regulators of lymphangiogenesis, which trigger LEC proliferation, migration and sprouting, while simultaneously promoting the maturation and stabilization of newly formed lymphatic vessels by modulating VE-cadherin-mediated intercellular junctions (Breslin et al., 2007; Tammela and Alitalo, 2010; Sung et al., 2022). Consistent with the functional phenotypes observed in LEC tube formation, thoracic duct ring culture and endothelial barrier assays, ELISA quantification verified that P-B-CM contained markedly higher concentrations of VEGF-C and VEGF-D relative to B-CM. Collectively, these data indicate that mechanical compressive stimulation remodels the paracrine secretion profile of BMSCs by upregulating the production of VEGF-C and VEGF-D, and these two cytokines serve as core signaling mediators that link mechanical microenvironmental cues to enhanced lymphangiogenic capacity of LECs.

Accumulating studies have explored the regulatory role of BMSCs in lymphangiogenesis, yet contradictory observations exist across published literature, which requires comprehensive and balanced interpretation. On one hand, multiple *in vitro* functional assays have validated that BMSC-derived conditioned medium exerts robust pro-lymphangiogenic activities, notably accelerating LEC proliferation, migratory capacity and tube-forming potential (Maertens et al., 2014; Robering et al., 2018). On the other hand, several independent investigations have documented neutral or negligible impacts of untreated BMSC secretions on LEC motility (Zhan et al., 2015). Multiple confounding variables jointly contribute to these inter-study discrepancies. First, the species origin of primary cells differs widely across experiments: rat, mouse and human BMSCs possess divergent secretory cytokine profiles, while LECs isolated from different tissue sources also exhibit distinct responsiveness to paracrine signals. Second, standardized protocols for conditioned medium collection have not been unified among groups, including variations in BMSC seeding density, culture duration, serum concentration and medium volume for supernatant harvesting. Third, the baseline culture microenvironment of BMSCs, such as static culture without mechanical stimulation, limits their intrinsic pro-lymphangiogenic secretory activity, leading to weak or undetectable phenotypes in some control groups. Consistent with the pro-lymphangiogenic phenotype observed in our cell and ex vivo tissue models, our data confirm that unstimulated BMSCs can positively regulate multiple LEC biological functions covering proliferation, migration and tube assembly. More importantly, our study further clarifies that compressive mechanical force significantly potentiates this beneficial paracrine regulatory effect of BMSCs. This mechanical priming largely eliminates the weak baseline effect of resting BMSCs reported in prior conflicting literature. Our finding aligns with prior mechanobiology reports stating that compressive loading reshapes the pro-angiogenic secretome of BMSCs (Shen et al., 2022), and fluid shear stress serves as a vital modulator of LEC migratory behavior (Surya et al., 2016). Collectively, this work expands the existing knowledge of how mechanical microenvironments govern lymphovascular remodeling within bone defect repair microenvironments.

Despite the significant findings presented in this study, several unavoidable limitations should be acknowledged. First, all cellular compression experiments were conducted via a static pressurization device rather than dynamic bioreactors. This static loading model cannot fully mimic the complex, time-varying mechanical stimuli occurring in native bone tissue microenvironments. In addition, although we standardized the operating steps strictly before each experiment, regular equipment calibration and quantitative detection of pressure distribution uniformity were not performed in this work. Uneven pressure transmission across the cell culture surface may introduce subtle differences in cellular responses. This technical shortcoming is a widespread challenge for *in vitro* mechanobiology research, and our current data can lay a solid foundation for subsequent research adopting precisely calibrated dynamic bioreactors with uniform pressure output. Second, this study mainly focused on detecting the secretion levels of VEGF-C and VEGF-D to confirm the paracrine effects of compressed BMSCs. Broad-spectrum secretome profiling and downstream intact molecular signaling cascades triggered by mechanical stimulation were not systematically explored. The precise intracellular regulatory axes linking compressive force, BMSC secretory phenotype and enhanced lymphangiogenesis of LECs remain unclear and require comprehensive verification in our follow-up mechanistic investigations.

## 5 Conclusions

Taken together, this study demonstrates for the first time that appropriate physiological mechanical pressure preconditioning can effectively activate BMSCs and enhance their paracrine-mediated pro-lymphangiogenic potential. At both cellular and tissue levels, we confirmed that conditioned medium from pressure-preconditioned BMSCs significantly promotes the proliferation, migration and tube formation of LECs. These findings not only expand our understanding of mechanobiological regulation of stem cell function and lymphatic tissue regeneration, but also provide novel experimental evidence and a promising therapeutic strategy for lymphatic system injury repair, particularly in the field of oral and maxillofacial tissue reconstruction. Future studies will focus on elucidating the detailed downstream molecular signaling pathways and validating the therapeutic efficacy in dynamic *in vivo* mechanical microenvironments.

## Data Availability

The original contributions presented in the study are included in the article/supplementary material. Further inquiries can be directed to the corresponding author.
